# Rare Presentation of Tuberculosis Mimicking Malignancy

**DOI:** 10.7759/cureus.20841

**Published:** 2021-12-30

**Authors:** Niraj K Choudhary, Rudra P Samanta, Rinku Bhargava

**Affiliations:** 1 Department of Neurosurgery, Tata Main Hospital, Jamshedpur, IND; 2 Department of Pulmonology, Tata Main Hospital, Jamshedpur, IND; 3 Department of Neurological Surgery, Tata Main Hospital, Jamshedpur, IND

**Keywords:** anti-tuberculosis therapy, mediastinal lymphadenopathy, malignancy, metastasis, tuberculosis

## Abstract

Tuberculosis is one of the most common infectious diseases that has a varied presentation and can affect most of the organs of the body. Differentiating disseminated tuberculosis with minimal symptoms from malignancy is difficult and makes the diagnosis challenging. No single test can be earmarked to diagnose tuberculosis and multiple investigations are mostly required. Here, we would like to discuss a rare case of a young male with mild symptoms who, on initial investigations, was provisionally diagnosed as having a metastatic disease, but detailed investigations proved it to be a case of disseminated tuberculosis. Through this case, we learn that radiological images alone cannot help in diagnosing even a common disease like tuberculosis. A complete workup helped in differentiating malignancy from tuberculosis in this mildly symptomatic patient.

## Introduction

Tuberculosis (TB) is the commonest infectious disease in the adult population worldwide and is associated with huge morbidity and mortality [1]. The number of deaths reported each year due to tuberculosis is approximately 1.3 million [2]. The most common sites of TB are the lungs, and in extrapulmonary sites, the vertebral column remains the most affected site. This disease continues to be a major health problem in India. Even with evolving health technology and advances in diagnostic modalities, the diagnosis of TB remains a problem area for clinicians. Due to the multifocal involvement and varied symptomology, clinicians need a multi-diagnostic approach that includes blood investigations, radiological imaging, and culture growth of *Mycobacterium tuberculosis *(MTB) [2]. Disseminated tuberculosis involving multiple organs with negligible symptoms is rare. On radiological images where multiple sites of involvement are seen, it becomes difficult to differentiate tuberculosis from metastatic lesions. Herein lies the significance of multiple diagnostic modalities and culture reports for making the final diagnosis. We report a case of multifocal tuberculosis diagnosed with a combination of multiple methods. The unusual sites of involvement with minimal symptomology made this case rare and challenging.

## Case presentation

A 19-year-old male presented with a low-grade fever for four to five days, associated with occasional mild and ill-defined backaches with no neurological deficit. The fever was intermittent, with a temperature range of 99.2 to 99.8 °C and no diurnal variation. There was no history of prolonged fever, cough, breathing difficulty, loss of appetite, bony pains, hemoptysis, or weight loss. He had no significant past medical or surgical history. There was no family history of tubercular infection.

**Figure 1:**
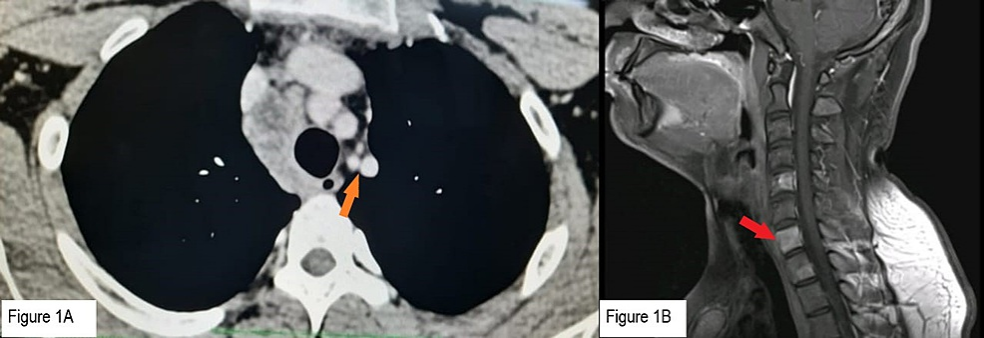
Radiological images before treatment. (A) Orange arrow: CECT thorax showing mediastinal lymphadenopathy. (B) Red arrow: CEMRI Spine showing contrast-enhancing lesions in vertebrae. CEMRI: contrast-enhanced magnetic resonance imaging, CECT: contrast-enhanced computed tomography.

With this not so significant symptomatology, he was investigated. His total and differential blood counts were normal. ESR was 35 mm/h and CRP was 5.1 mg/dl. A chest X-ray showed normal lung parenchyma with a doubtful para tracheal opacity. In view of raised CRP and doubtful lymphadenopathy, a contrast-enhanced computed tomography (CECT) thorax and abdomen were planned. The CECT thorax showed multiple necrotic prevascular and pretracheal conglomerated necrotic mediastinal nodes, with the largest measuring 18 mm (Figure 1A).

The CECT abdomen showed a normal study. The CT findings were suggestive of tuberculosis, and hence, a Montoux test was also done. This was reported as positive after 72 hours.

In the meantime, in view of mild backache, a contrast-enhanced magnetic resonance imaging (CEMRI) dorso lumbar spine with screening was done, which changed the whole diagnostic picture. It showed multifocal areas of hyper-intense lesions with post-contrast enhancement in multiple vertebrae involving the vertebral bodies, pedicles, and posterior spinal elements (Figure 1B). Altered marrow signal intensity was also noted in the bilateral iliac bones and right-sided lower ribs. No associated soft tissue involvement was seen. Disc spaces were maintained. Seeing wide and multiple bony lesions, a strong suspicion of bony metastasis was made and communicated to the family. Other suggestive differentials were lymphoma and multiple myeloma. A complete blood picture along with serum protein electrophoresis was done, which was normal. Serum protein, along with albumin globulin ratio, was also normal, ruling out the possibility of multiple myeloma.

**Figure 2:**
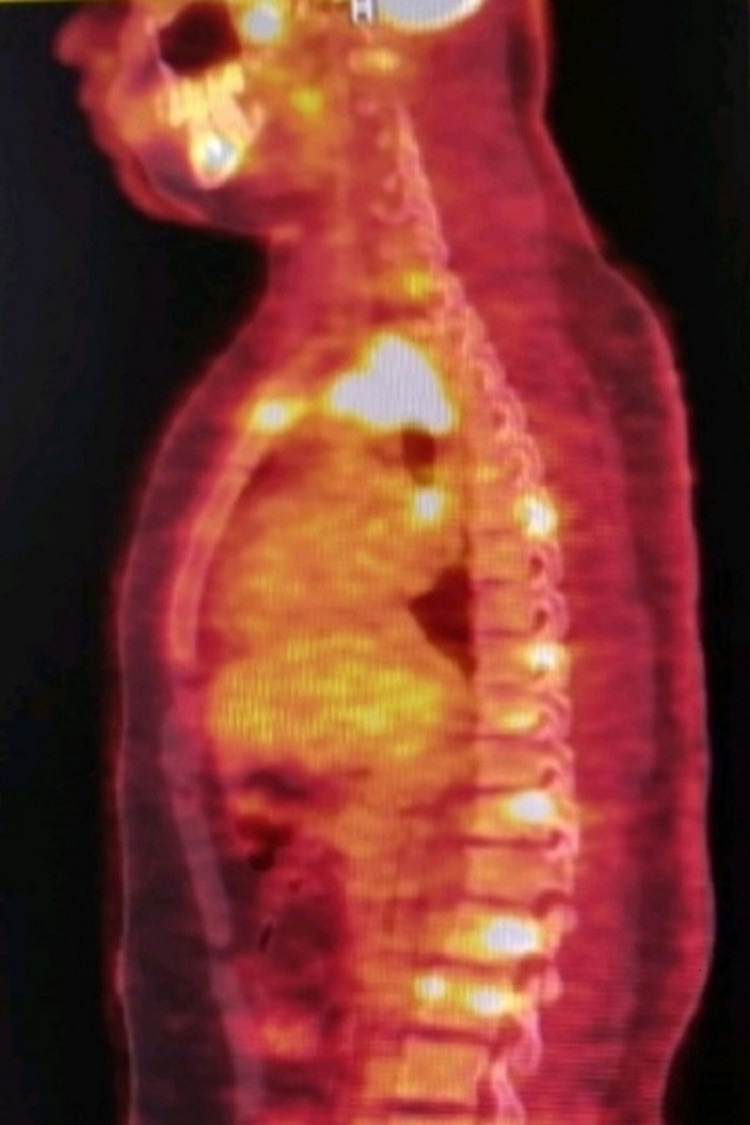
PET CT before treatment. Image showing increased FDG uptake at multiple bony sites.

The next modality of investigation was PET CT, which was used to determine the primary site of the lesion as well as the extent of disease spread. This showed multiple areas of marrow F-18 fluoro deoxyglucose (FDG) uptake in the visualized skeleton, predominantly involving anterior and posterior elements of multiple cervico-dorsal and lumbar vertebra, bilateral iliac bones, sacrum, left scapula, medial end of right clavicle, sternum, and bilateral ribs (Figure 2).

At this stage, TB was the last possibility in mind because of extensive bony involvement and atypical sites of vertebral involvement. The distinction of neoplastic deposits from an unknown primary or lymphoma was proposed.

To confirm the diagnosis of the disease, an endobronchial ultrasound (EBUS) guided biopsy of the mediastinum lymph nodes was carried out. The Gene X-pert study from the biopsy sample revealed MTB and a rifampicin sensitive strain. The EBUS transbronchial needle aspiration features were suggestive of granulomatous inflammation favoring tuberculosis. The endoscopic biopsy was reported as a single epithelioid granuloma. However, the CT-guided bone biopsy from the sternum showed no granuloma or malignancy. After a battery of investigations, finally a diagnosis of disseminated tuberculosis was made, involving mediastinal lymph nodes and multiple bony sites, which are very rare in TB.

Based on the diagnosis, the patient was treated conservatively with first-line anti-tubercular drugs, which included Rifampicin (600 mg/day), Isoniazid (300 mg/day), Pyrazinamide (2000 mg/day), and Ethambutol (1600 mg/day), along with Pyridoxine (40 mg/day). Within five days of starting treatment, the fever subsided. After one month of treatment, both CRP and ESR came down to 0.82 mg/dl and 22 mm/h, respectively. His liver function tests were within normal limits, so the same drug regimen was continued. A pending culture report from the mediastinal lymph node showed growth of M. tuberculosis complex by the Bactec system. This further confirmed the diagnosis of TB.

After two months of treatment, CRP became 0.47 mg/dl and ESR came to 3 mm/h (Table 1).

**Table 1:** Serial ESR and CRP values.

Investigation	ESR (mm Ist hour)	CRP (mg/dl)
Initial	35	5.15
After 1 week of ATT	44	3.57
After 1 months of ATT	22	0.82
After 2 months of ATT	03	0.47

**Figure 3:**
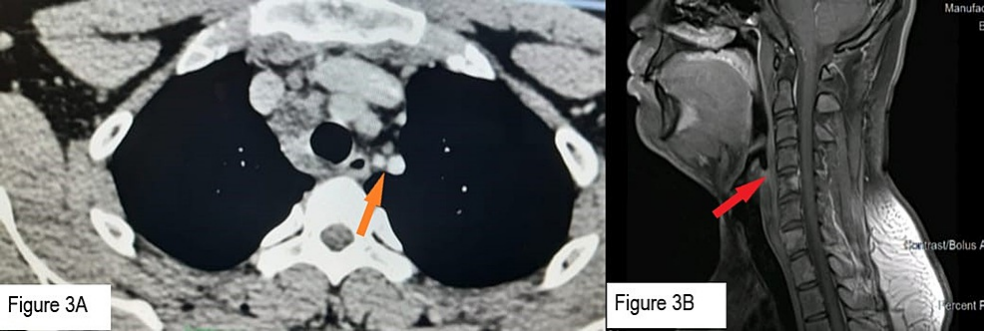
Radiological images after two months of treatment. (A) Orange arrow: CECT thorax showing decrease in size of mediastinal lymph nodes. (B) Red arrow: CEMRI spine showing decreased contrast uptake in vertebrae.

The tablet pyrazinamide was stopped, and the patient continued the remaining three drugs. A CECT thorax was done, which showed a decrease in the size of lymph nodes (Figure 3A). CEMRI of the dorso-lumbar spine with screening of the whole spine was also done, showing a significant decrease in the size of bony involvement suggestive of healing of the bony lesions (Figure 3B).

Clinically patient remained asymptomatic.

## Discussion

Tuberculosis is a very common disease in India, with significant morbidity and mortality. Amongst the extra pulmonary tuberculosis sites, the mediastinal lymph nodes and the vertebral column are the most commonly involved sites [3]. Diagnosis becomes a challenge when the investigation results done for TB simulate malignancy [4]. In this case also, the initial clinical investigations and symptomology gave no clue towards TB and later on mimicked malignancy. As per WHO, radiological screening plays an important role in the diagnosis of TB [2]. Here, the findings of the MRI spine changed the whole diagnosis and shifted it towards malignancy. The next investigation of choice was PET CT, which differentiates between normal and malignant cells due to the increased metabolic uptake of F-18 FDG. An increased uptake was found in PET imaging, which still left us in a diagnostic dilemma. The main drawback of this test is its false positivity at times, as it shows increased uptake even with inflammatory cells [5,6]. As is evident here, even various radiological investigations could not differentiate between malignancy and tuberculosis. The patient presented with multiple bony lesions, including vertebrae and mediastinal lymph nodes. Various literature suggests that lymph node involvement is very common both in tuberculosis and in malignant diseases [7]. Mediastinal lymph nodes were chosen for biopsy due to easy access and for diagnostic confirmation. The histopathology report showed granulomatous inflammation and the polymerase chain reaction (PCR) test (Xpert MTB/RIF) proved the existence of MTB and clinched the diagnosis of TB. We were fortunate to reach the diagnosis of TB from the biopsy, but it must be noted that a decreased bacterial load in the lymph nodes could also lead to a negative result. Subsequently culture report (BACTEC system) was also positive which is considered as the gold standard for diagnosis for MTB. Finally, we could reach to a conclusive diagnosis of TB with the help of multiple investigative modalities.

## Conclusions

The present case enlightened us on the varied and rare presentations of tuberculosis, which can mimic malignancy. The presence of extensive disease with minimal symptomatology was the highlight of this case. It also signified the requirement for multiple diagnostic measures at times to diagnose a common disease such as tuberculosis. Timely diagnosis and proper management with strict drug compliance played a major role in preventing the patient from severe morbidity associated with disseminated tuberculosis and ensured a good prognosis. It also carried a message for the clinicians that they need to be doubly sure with adequate investigation reports before disclosing any bad news like cancer to the family members.
